# Acid Ceramidase Maintains the Chondrogenic Phenotype of Expanded Primary Chondrocytes and Improves the Chondrogenic Differentiation of Bone Marrow-Derived Mesenchymal Stem Cells

**DOI:** 10.1371/journal.pone.0062715

**Published:** 2013-04-26

**Authors:** Calogera M. Simonaro, Sylvain Sachot, Yi Ge, Xingxuan He, Victor A. DeAngelis, Efrat Eliyahu, Daniel J. Leong, Hui B. Sun, Jeffrey B. Mason, Mark E. Haskins, Dean W. Richardson, Edward H. Schuchman

**Affiliations:** 1 Genetics & Genomic Sciences, Mount Sinai School of Medicine, New York, New York, United States of America; 2 Orthopaedic Surgery Albert Einstein College of Medicine, Bronx, New York, United States of America; 3 Radiation Oncology, Albert Einstein College of Medicine, Bronx, New York, United States of America; 4 Clinical Studies, University of Pennsylvania School of Veterinary Medicine, New Bolton Center, Kennett Square, Pennsylvania, United States of America; 5 Pathobiology, University of Pennsylvania School of Veterinary Medicine, Philadelphia, Pennsylvania, United States of America; University of Duisburg-Essen, Germany

## Abstract

Acid ceramidase is required to maintain the metabolic balance of several important bioactive lipids, including ceramide, sphingosine and sphingosine-1-phosphate. Here we show that addition of recombinant acid ceramidase (rAC) to primary chondrocyte culture media maintained low levels of ceramide and led to elevated sphingosine by 48 hours. Surprisingly, after three weeks of expansion the chondrogenic phenotype of these cells also was markedly improved, as assessed by a combination of histochemical staining (Alcian Blue and Safranin-O), western blotting (e.g., Sox9, aggrecan, collagen 2A1), and/or qPCR. The same effects were evident in rat, equine and human cells, and were observed in monolayer and 3-D cultures. rAC also reduced the number of apoptotic cells in some culture conditions, contributing to overall improved cell quality. In addition to these effects on primary chondrocytes, when rAC was added to freshly harvested rat, equine or feline bone marrow cultures an ∼2-fold enrichment of mesenchymal stem cells (MSCs) was observed by one week. rAC also improved the chondrogenic differentiation of MSCs, as revealed by histochemical and immunostaining. These latter effects were synergistic with TGF-beta1. Based on these results we propose that rAC could be used to improve the outcome of cell-based cartilage repair by maintaining the quality of the expanded cells, and also might be useful *in vivo* to induce endogenous cartilage repair in combination with other techniques. The results also suggest that short-term changes in sphingolipid metabolism may lead to longer-term effects on the chondrogenic phenotype.

## Introduction

Cell-based therapy for cartilage repair has gained increasing popularity since the first reports of successful autologous chondrocyte implantation (ACI) over 10 years ago [1]. In ACI, primary chondrocytes are obtained from small biopsies of healthy articular cartilage, expanded, and then placed onto 3-D scaffolds for subsequent use in cartilage repair surgery (for review see [2]). Currently, ACI is used in ∼10% of all cartilage repair procedures where the lesions are less than 2–4 cm^2^ [3]. ACI also has been used in veterinary medicine to improve the outcome of cartilage repair surgery in large (equine) and small (dog) animals [4], [5]. There have been many reports documenting the improved clinical effectiveness of ACI as compared to other cartilage repair procedures, and several large, multi-site clinical studies are currently underway [6]. However, an important limitation of this procedure is the requirement of two invasive surgeries, the first of which requires extraction of cells from healthy cartilage tissue, and the second to implant the cells that have been expanded *ex vivo*. Recent research has therefore focused on the use of alternative chondrocyte sources where the cells can be obtained less invasively (e.g., nasoseptal) [7], the generation of chondrocytes from adult stem cells (e.g., mesenchymal stem cells [MSC] from the bone marrow or adipose tissue), and/or the use of MSCs directly for transplantation [8]–[10].

A key factor in the development of any cell-based therapy is to find safe and effective methods to rapidly expand autologous cells in a manner that retains their phenotype and *in vivo* repair potential. For ACI, research has concentrated on defining the culture media and growth factors used for articular chondrocyte expansion, as well as the improved design and formulation of scaffolds used to adhere the cells and prepare them for surgical re-implantation. Currently, most culture medias used to expand primary articular chondrocytes contain serum supplemented with growth factors, including members of the transforming growth factor (TGF-beta1–3) and bone morphogenic families (BMPs), insulin growth factor 1 (IGF1), fibroblast growth factor 2 (FGF2) and others (for review see [11]). Similarly, numerous transcription factors influence chondrogenesis, including Sox9, beta-catenin, Smads and others, resulting in optimal expression of chondrocyte-specific markers. Sox9 in particular is required for pre-cartilage condensation and differentiation of chondroprogenitor cells into chondroblasts [12].

Several studies have demonstrated the importance of sphingolipid signaling on cartilage homeostasis. For example, an early report [13] showed that a synthetic ceramide derivative (C2 ceramide) stimulated the expression of matrix metalloprotease (MMP) −1, 3 and 13 in rabbit articular chondrocytes, and induced chondrocyte apoptosis. Gilbert et al [14] also showed that treatment of bovine articular chondrocytes with sphingomyelinase, an enzyme that produces ceramide by sphingomyelin hydrolysis, decreased expression of collagen II. Elevated ceramide also has been documented in patients with rheumatoid and osteoarthritis [15], and inhibition of sphingosine-1-phosphate (S1P) production in induced rodent models of arthritis has led to beneficial clinical results [16].

In addition, we have found that animals with genetic deficiencies of enzymes involved in glycosaminoglycan degradation (i.e., the mucopolysaccharidoses, MPS) have numerous abnormalities in sphingolipid metabolism in their connective tissues. For example, chondrocyte apoptosis and cartilage degradation in the MPS animals is associated with elevated ceramide, while synovial hyperplasia is associated with elevated S1P [17]. Elevated AC activity also can be detected in serum and synovial fluid from MPS animals, likely a response to the elevated ceramide (unpublished finding).

In the current study the use of rAC as a media supplement to improve the quality of expanded primary and MSC-derived chondrocytes for cartilage repair was evaluated. Rat, equine and human articular chondrocytes treated once with rAC in either monolayer or 3-D culture systems had an improved chondrogenic phenotype after 2–3 week cell expansion, including enhanced expression of Sox9, FGF2, collagen 2, aggrecan and other chondrogenic markers, and decreased detection of collagen 10. In addition, rat, feline and equine bone marrow cells grown for only 1 week in the presence of rAC yielded ∼2-fold more MSCs, and rAC treatment also led to enhanced chondrogenesis of MSCs. We propose that rAC may be an important supplement to include in chondrocyte and MSC growth media that improves the production and chondrogenic potential of cells used for cartilage repair.

## Materials and Methods

### Animals

Animals were raised under National Institutes of Health and USDA guidelines for the care and use of animals in research. All animal protocols were approved by the Mount Sinai Institutional Animal Care and Use Committee (protocol # 98–0108), and were performed in accordance with NIH guidelines. Normal rats were identified by genotyping from the MPS VI breeding colony [18] maintained at the Mount Sinai School of Medicine, while the larger animals (cats and horses) were maintained at the University of Pennsylvania School of Veterinary Medicine (Philadelphia and New Bolton Center, PA, USA, respectively). The animals were housed with *ad libitum* food and water. Euthanasia was performed on cats using 80 mg/kg of sodium pentobarbital (Veterinary Laboratories, Lenexa, KS, USA) in accordance with the American Veterinary Medical Association guidelines. Euthanasia of rats was performed using carbon dioxide inhalation. Equine cells were obtained during routine surgical procedures.

### Primary Chondrocyte Isolation and Culture

Methods for rat articular chondrocyte isolation and culture have been published elsewhere [19]. Briefly, to establish rat primary chondrocyte cultures, femoral caps and meniscus were collected from 6 age and sex-matched adult animals and washed three times in 0.9% NaCl supplemented with 5% penicillin/streptomycin (v/v) and fungizone 0.1% (v/v). The cartilage was digested with 0.5 mg/ml pronase prepared in 0.9% NaCl (v/v) +5% penicillin/streptomycin (v/v) +0.1% fungizone for 2 h at 37°C, and with 1 mg/ml collagenase (Sigma Chemical Co.) prepared in DMEM containing 10% FBS (v/v), 1% penicillin/streptomycin (v/v) and 0.1% fungizone overnight at 37°C. The resulting media was then passed through a 40 µm cell strainer, and the cells were isolated by centrifugation (1200 rpm for 8 min). They were further washed twice in DMEM containing 10% FBS, and plated in 75 cm^2^ flasks at a density of 7000 cells per cm^2^ in DMEM containing 10% FBS (v/v), 1% penicillin/streptomycin (v/v) and fungizone 0.1%. The freshly plated cells were considered passage 0 (P0). When reaching subconfluency, cells were trypsinized and passaged (P1) for further expansion. For subsequent passages, the cells were grown for 7 days, trypsinized, and then re-seeded at the same cell density. In some experiments rAC was added to the media at P0 (200 U/ml final concentration). Unless otherwise noted rAC was added to the primary chondrocytes only once, and subsequent media changes did not include the enzyme.

To grow rat chondrocytes in 3-D cultures, 5×5 mm collagen sponges (CollaCote, Zimmer Dental Inc., Carlsbad, CA, USA) were used. The sponges were pre-wet in DMEM containing 10% FBS (v/v) and antibiotics, and the cells, which had been previously isolated as described above, were suspended in media and pipetted onto the sponge. To distribute the chondrocytes throughout the sponge it was compressed slightly to circulate the media and distribute the cells. The sponges were then placed in culture dishes and grown in DMEM containing 10% FBS and antibiotics with and without rAC. The media was changed every three days with fresh media containing rAC (200 U/ml).

To grow rat chondrocytes in fibrin gels, 7.5×10^6^ cells were seeded in 200 µl of TISSEEL® (Baxter Healthcare, Deerfield, IL, USA) gel in 96 well plates and cultured in standard media (DMEM containing 10% FBS and antibiotics) with or without rAC (200 U/ml). Media was changed every 4 days with fresh rAC included each media change. Cells were maintained for 2 weeks prior to analysis.

To obtain equine articular chondrocytes, cartilage explant tissue was recovered from the articular surfaces of the stifle joints of geldings (age 3–12 years) by use of a 6-mm^2^ biopsy punch. The punch was pushed into the cartilage perpendicular to the articular surface until contact was made with the surface of the bone. The punch was then rotated and removed, and the resultant explant was separated from the bone by use of a scalpel blade via dissection parallel to the articular surface. Cartilage explants were trimmed to a thickness of approximately 100 µm to eliminate any mineralized tissue that might have affected subsequent culture or analysis procedures. Chondrocytes were isolated from explant tissue as described elsewhere [20], and either frozen (P0) or used for cell expansion in DMEM/F12 media containing 50 µg of ascorbate-2-phosphate/ml (v/v), 1% (v/v) penicillin/streptomycin, 1% (v/v) glutamate and 0.1% (v/v) fungizone.

Human articular cartilage was collected from patients undergoing total knee replacement surgery with written consent in accordance with the Institutional Review Board of the Mount Sinai Program for the Protection of Human Subjects (IRB-approved protocol #09-0248). Cartilage slices were collected into DMEM/F12 media without FBS (containing 1% (v/v) penicillin/streptomycin, 1% (v/v) glutamate and 0.1% (v/v) fungizone), and then incubated with Pronase (1 mg/ml) in DMEM/F12 containing 10% FBS (v/v) for 30 min at 37°C in a shaker. Following centrifugation at 200×g, the cartilage was washed twice with PBS and then incubated with collagenase P (1 mg/ml) in DMEM/F12 containing 10% FBS (v/v) overnight at 37°C in a shaker. The cartilage was then filtered using a 40–70 µm filter, centrifuged and the supernatant was discarded. The cells were washed once with PBS, resuspended in DMEM/F12 media containing 10% FBS (v/v), and plated at a density of 1×10^5^/cm^2^. In some experiments rAC was added to the culture media immediately at the time of cell plating (200 U/ml final concentration).

### Acid Ceramidase, Ceramide, Sphingosine and Sphingosine-1-phospate Measurements

Rat chondrocytes were grown in 12-well plates with or without rAC (200 U/ml), and AC activity, ceramide, sphingosine and sphingosine-1-phosphate levels were determined in cell lysates at 12, 24 and 48 h. Cells were harvested and total proteins were extracted using the Cell Lytic^TM^M Cell Lysis Reagent (Sigma, Saint Louis, MO, USA). Proteins were quantified (Bio-Rad, Hercules, CA, USA), and the lysates were subjected to AC enzymatic activity measurement as described previously [21]. Lipids also were extracted from the cell lysates, and ceramide, sphingosine and sphingosine-1-phosphate levels were quantified as already published [22], [23].

### Mesenchymal Stem Cell Isolation and Culture

Rat MSCs were obtained from the femoral and tibia cavities of adult rats. Femurs and tibias were isolated, the extremities were removed, and the bone marrow was flushed out using PBS. After 2 washings in PBS, bone marrow cells were counted and plated at a density of 5×10^6^ cells/cm^2^ in Alpha MEM supplemented with 20% (v/v) FBS, 1% (v/v) penicillin/streptomycin, 1% (v/v) glutamate and 0.1% (v/v) fungizone. Bone marrow from cats was collected during post mortem procedures and shipped in transport medium (RPMI 1640, 1% (v/v) penicillin/streptomycin, 1% (v/v) L-glutamine) overnight.

For horses, bone marrow was collected aseptically from the sternum by use of an 11-gauge bone marrow biopsy needle and a 60 ml sterile Luer-tip syringe that contained 10 ml of sterile acid-citrate-dextrose solution. After aspiration of the bone marrow, the aspirate was briefly mixed with the acid-citrate-dextrose solution, and the syringe then was placed on ice and shipped overnight. Once received, cells were washed twice with PBS and plated at a density between 1–5×10^6^ cells/cm^2^ (feline cells) and 3×10^8^ cells/cm^2^ (equine cells) in Alpha MEM supplemented with 20% (v/v) FBS, 1% (v/v) penicillin/streptomycin, 1% (v/v) glutamate and 0.1% (v/v) fungizone. The freshly plated bone marrow cells were treated with or without rAC at day 0 (200 U/ml final concentration). At day 3, medium was removed and replaced by fresh medium lacking rAC. Medium was subsequently changed twice a week, and cells were passaged when they reached subconfluency.

In some experiments the cells were stained after one week of culture with crystal violet (0.5% [Sigma] w/v in methanol) to count the number of colonies. For these assays (CFU-F) cells were incubated 30 min at room temperature and rinsed 4 times with PBS, before a final washing in water. Only colonies with a diameter greater than 1 mm were counted.

In experiments using rat cells, flow cytometric analysis also was performed to assess the number of MSCs. For these analyses MSCs were collected, washed twice in PBS supplemented with 2% FBS and marked for CD90-FITC and CD45-PE (Cat# 11-0900-81 and Cat# 554878, respectively, BD Biosciences Pharmingen, San Diego, CA, USA) diluted in PBS+FBS 2% for 15 min on ice and in the dark. After subsequent washings, cells were analyzed in a LSR II flow cytometer (BD Biosciences, San Jose, CA, USA). Flow cytometry analysis could not be performed on the cat and horse cells due to the lack of suitable antibody reagents.

To test the effect of rAC on the chondrogenic potential of BM-MSCs, bone marrow cells were amplified for 3 weeks in Alpha MEM supplemented with 20% (v/v) FBS, 1% (v/v) penicillin/streptomycin, 1% (v/v) glutamate and 0.1% (v/v) fungizone. rAC was not included during this cell expansion phase. At the end of 3 weeks the cells were trypsinized, counted, and 5×10^5^ cells were differentiated in pellet cultures using conical culture tubes. Chondrogenic differentiation was performed in DMEM high glucose media containing 6.25 µg/ml insulin, 6.25 µg/ml transferin, 1.25 mg/ml bovine serum albumin, dexamethasone 100 nM, ascorbate-2-phosphate 50 µM, 5.33 µg/ml linoleic acid and 10 ng/ml TGF-beta1, as described previously [24]. To evaluate the effect of rAC on the chondrogenic differentiation, in some experiments rAC was added into the chondrogenic medium (200 U/ml final concentration). Medium was changed every 3 days, with or without rAC. Cells were maintained in a humidified incubator at 37°C under 5% CO_2_.

### Recombinant AC Production and Purification

Human rAC was produced in Chinese hamster ovary (CHO) cells as described previously [25]. Briefly, rAC overexpressing CHO cells were grown to confluency in DMEM supplemented with 10% v/v FBS and 1% penicillin/streptomycin. The conditioned medium was collected, and rAC was concentrated by pressure filtration (cut off 30 kDa, Amicon, Billerica, MA, USA) and purified using a fast protein liquid chromatography system (Amersham Biosciences, Piscataway, NJ, USA). The amount of rAC was quantified by enzyme activity measurement and western blot analysis as previously described [21], [26].

### Processing of the Differentiated Mesenchymal Stem Cell Pellet Cultures

Once BM-MSCs were differentiated in the chondrogenic media with or without rAC, pellets were removed from the incubator, washed twice, and fixed in paraformaldehyde 4% for 15 min at room temperature. They were then dehydrated by successive incubations in 70% ethanol (30 min at room temperature), 95% ethanol (2×30 min at room temperature) and 100% ethanol (2×30 min at room temperature). Pellets were then cleared in two successive baths of xylene (2×30 min at room temperature), and paraffin embedded and microtome processed to create 6 µm slices on polyprep slides (Sigma).

For histochemical staining, pellets were deparaffinized in xylene, hydrated with distilled water, and stained with either Alcian Blue 8GX 1% (w/v), pH 2.5 (Sigma) for 30 min at room temperature or Safranin-O (Sigma) 0.1% (w/v) for 5 min at room temperature. Slides were washed, dehydrated and cleared with xylene before covering with mounting medium.

### Immunohistochemistry

For immunohistochemical analysis pellets were deparaffinized in xylene, hydrated and washed three times in PBS. If needed (i.e., for Sox9 immunostaining), cells were permeabilized in 0.2% (v/v) Triton X-100 made in PBS (pH 7.4) for 5 min at room temperature, post-fixed in paraformaldehyde, and washed in PBS several times. Slides were then blocked 2 h at room temperature in PBS supplemented with Tween 0.1% (v/v), and 10% (v/v) FBS. After blocking, slides were incubated with primary antibody diluted in PBS containing 0.1% Tween and 5% FBS overnight at 4°C. The following primary antibodies from Santa Cruz Biotechnology, Santa Cruz, CA, USA were used for immunostaining: Rabbit anti-Sox9 (H-90, sc-20095), rabbit anti-collagen 2A1 (H-300, sc-28887), goat anti-collagen 10A1 (E-14, sc-323750), and goat anti-aggrecan (D-20, sc-16492). After exposure to the primary antibodies, slides were then incubated 1 h at room temperature with the corresponding secondary antibody conjugated with Cy3, diluted in PBS/Tween 0.1%/10% FBS. Finally, the slides were washed several times in PBS, and mounted with a DAPI containing medium (Vector Laboratories, Burlingame, CA, USA). Localization of the primary antibodies was visualized using the fluorescent Cy-3 second antibody and laser-scanning confocal microscopy (Zeiss LSM510).

For immunohistochemistry of the primary chondrocyte cultures, P3 cells were plated on chamber slides (5000 cells/chamber, lab-tek2 chamber slide, Thermo Fisher Scientific, USA) and grown until subconfluency. Cells were then washed twice in PBS, fixed in paraformaldehyde 4% for 15 min at room temperature, and immunostained as described above.

### Western Blot Analysis

Primary chondrocytes were harvested by trypsinization, washed and the proteins extracted in Cell Lytic^TM^M Cell Lysis Reagent (Sigma, Saint Louis, MO, USA). Proteins were quantified (Bio-Rad, Hercules, CA, USA), samples were normalized for protein and analyzed by western blotting (Novex Protein Analysis Solutions, Invitrogen, Carlsbad, CA, USA). The following primary antibodies were used for western blot analysis from Santa Cruz Biotechnology, CA, USA. Rabbit anti-Sox 9 (H-90, sc-20095), goat anti-collagen 1A2 (M-19, sc-8788), rabbit anti-collagen 2A1 (H-300, sc-28887), goat anti-aggrecan (D-20, sc-16492), mouse anti-FGF2 (sc-135905), rabbit anti-TGF-beta1 (sc146), mouse anti-Bax (2D2) (sc-20067), rabbit anti-PARP-1/2 (H-250) (sc-7150), rabbit anti-GAPDH (FL-335) (sc-25778), and goat anti-actin (C-11) (sc-1616). Rabbit anti-collagen 10 (ab58632) also was used from Abcam (Cambridge, MA, USA).

### RNA Isolation and Quantitative RT-PCR

For RNA isolation cells were trypsinized, washed twice in PBS, and resuspended in Trizol/chloroform (5/1 (v/v)) (Life Technologies, USA). After centrifugation, the aqueous upper phase was saved and purified through an affinity column (RNeasy Mini Kit, Qiagen, Valencia, CA, USA) according to the manufacturer’s instructions. RNA was resuspended in RNase-free water, quantified, and 1 µg of RNA was subjected to reverse transcription. For qPCR analysis of the human articular chondrocyte cultures, cDNA was synthesized with Superscript VILO (Invitrogen). SYBR green qPCR (Invitrogen Platinum Taq) was performed and gene expression was normalized to GAPDH (2-ddCt method).

### Statistical Analysis

Where appropriate, statistical analysis was carried out using a standard student’s t-test analysis, one-way analysis of variance (ANOVA) with the variable group, multivariate analyses of variance (MANOVAs) followed by post hoc Bonferroni adjustments. The results were considered significant at P<0.05. Statistics were performed using Sigma Stat 3.1 (Systat Software).

## Results

### Changes in Sphingolipid Metabolism Following rAC Treatment of Primary Articular Chondrocytes

**Figure 1:**
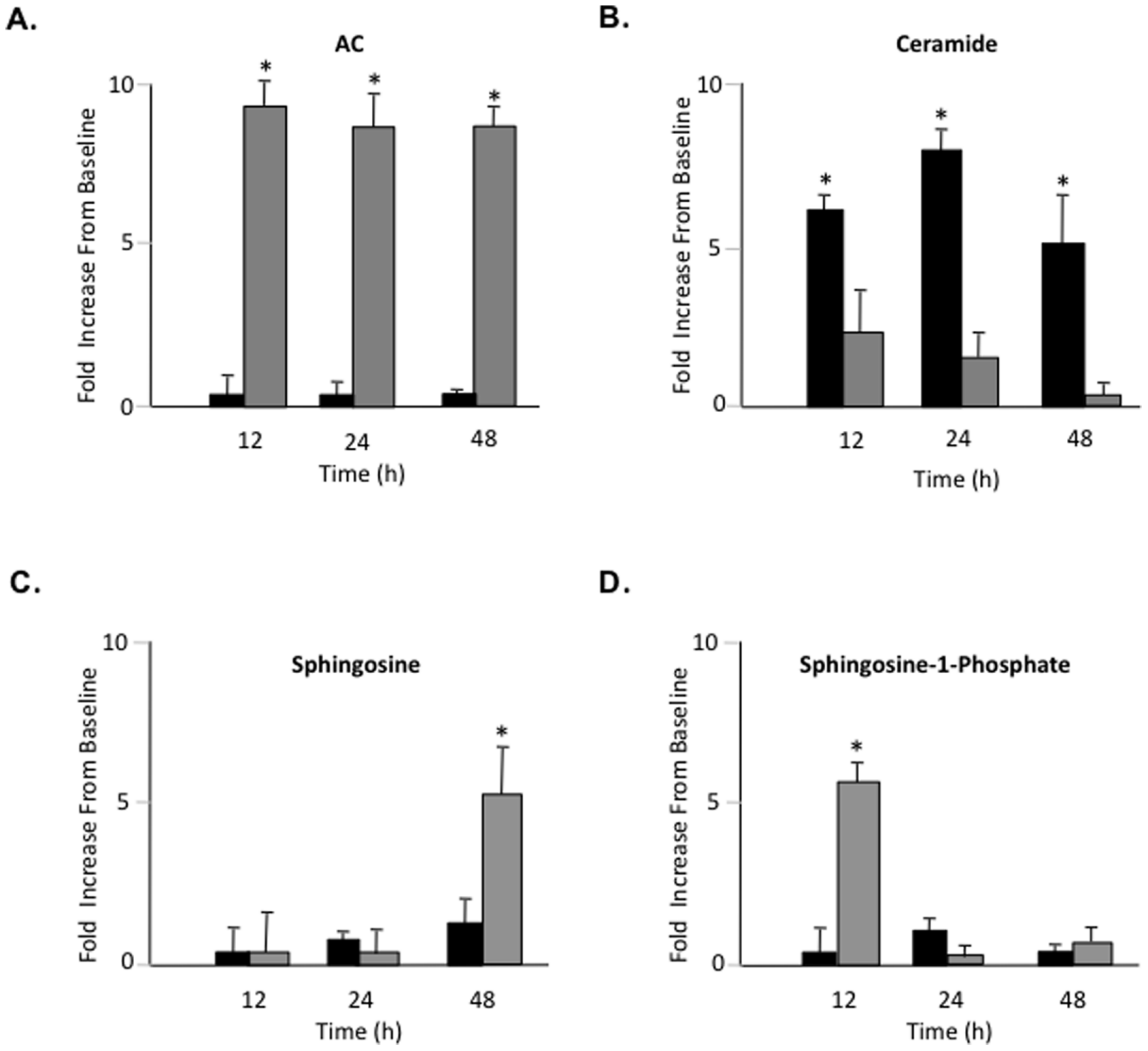
Primary rat articular chondrocytes were isolated and grown in monolayer cultures for 48 h with or without rAC (rAC, 200 U/ml) added to the media. At the indicated times proteins and lipids were extracted from the cells, and A) AC activity, B) ceramide, C) sphingosine, and D) sphingosine-1-phosphate levels were determined. Note that the addition of rAC to the culture media (gray bars) led to markedly increased AC activity in the cells by 12 h that was sustained for at least 48 h. In addition, by 12 h ceramide levels were elevated compared to baseline, but significantly less so in cells treated with rAC (i.e., by 48 h cells treated with rAC had no detectable ceramide elevation). Finally, the levels of S1P and sphingosine were elevated in rAC-treated cells by 12 and 48 h, respectively. * = p<0.05.

Primary rat articular chondrocytes were isolated and grown in monolayer cultures as described in the Materials and Methods using DMEM containing 10% FBS with or without rAC. For the baseline time point, cells were collected from 6 rats and pooled without culture. The AC activity, ceramide, sphingosine and sphingosine-1-phosphate (S1P) levels in these control cells were compared to cells grown for 12, 24 and 48 h with or without rAC. The data are summarized in Fig. 1.

As expected, cells grown in media supplemented with rAC exhibited markedly increased AC activity by 12 h, and this was sustained through 48 h. Ceramide levels also were increased compared to baseline by 12 h, but less in cells exposed to rAC. In fact, by 48 h the ceramide levels in the rAC-treated cells had returned to baseline, while in those without rAC treatment they remained significantly elevated. Surprisingly, despite the high ceramide levels we did not see elevation of sphingosine at 12 or 24 h. In contrast there was an early elevation of S1P, presumably due to the activity of sphingosine kinase. By 48 h significant elevations of sphingosine were detected in the rAC-treated cells. By 7 days AC, ceramide, sphingosine and S1P levels all had returned to baseline regardless of rAC treatment (data not shown).

Overall, these results demonstrated that rAC was taken up by rat chondrocytes and retained biological activity by hydrolyzing ceramide and producing sphingosine and S1P. The fact that AC levels returned to baseline by 7 days was consistent with its expected intracellular half-life of 48–72 h [27].

### Effects of rAC Supplementation on the Phenotype of Expanded Articular Chondrocyte Monolayer Cultures

We next evaluated the effects of rAC supplementation on the phenotype of articular chondrocytes after cell expansion. For these experiments rat chondrocytes were grown for 3 weeks with and without rAC in DMEM containing 10% FBS. rAC was added once at the time of the initial cell plating (P0). All analyses were performed at the end of the 3-week expansion period (P3) unless otherwise mentioned.

**Figure 2:**
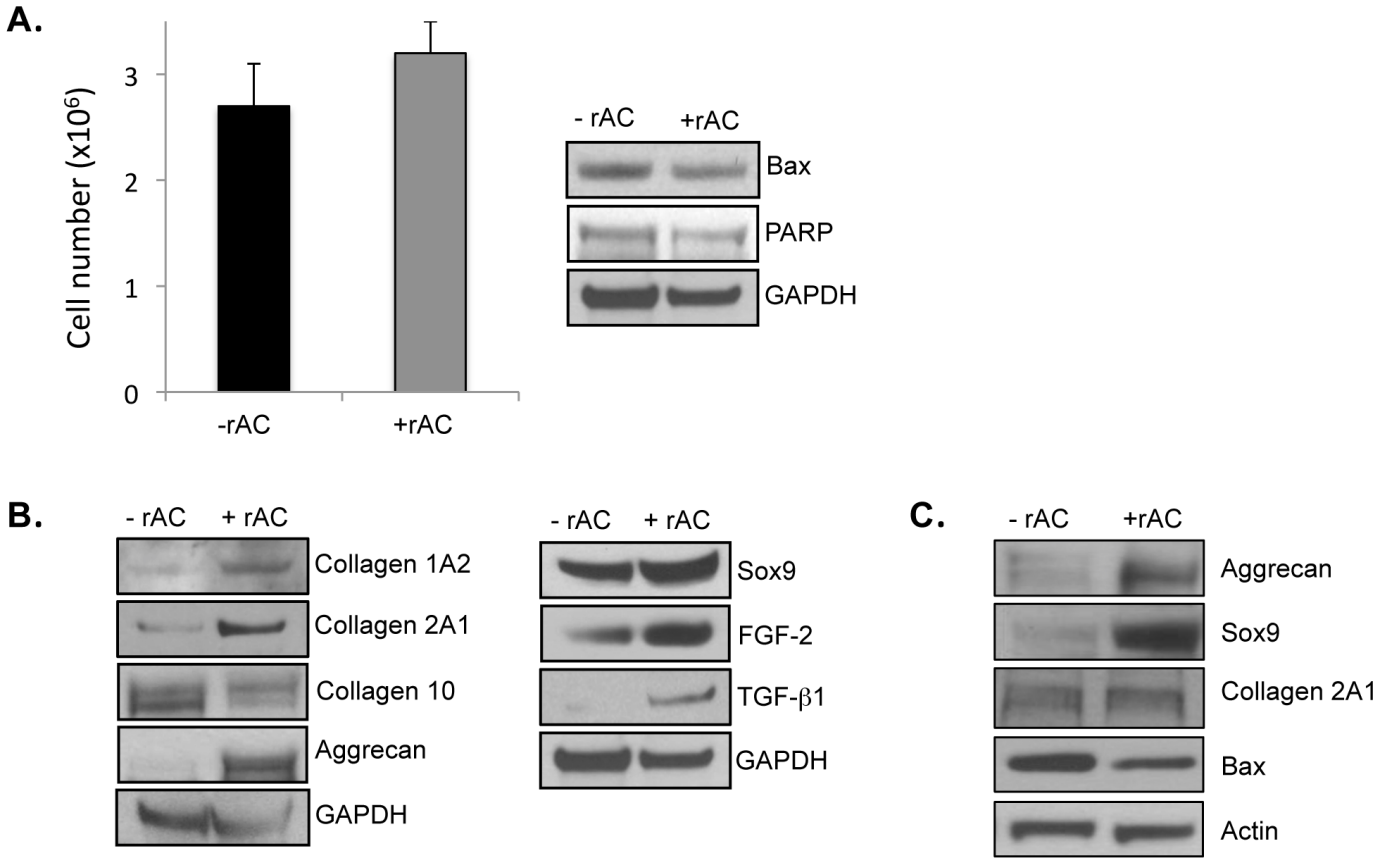
Rat articular chondrocytes were obtained from femurs and grown for 3 weeks in monolayer cultures using standard culture medium with or without recombinant human AC (rAC 200 U/ml). rAC was added once at the initiation of the cultures. At the end of the 3-week expansion period, the cells were harvested and analyzed. **A)** Total cell counts (left) revealed no differences in the presence of rAC. Western blotting for two apoptosis markers (Bax and PARP) similarly revealed no differences. **B)** The expanded chondrocytes were analyzed by western blots for several important chondrogenic markers, including collagens 1A2 and 2A1, aggrecan, Sox9, FGF2, and TGF-beta1. Note that in all cases these chondrocyte markers were elevated in the cells treated with rAC. In contrast to collagens 2A1 and 1A2, collagen 10 expression, a marker of hypertrophy, was lowered by rAC treatment. **C)** Horse articular chondrocytes were obtained surgically from femoral heads and frozen. The frozen cells were then recovered and grown in monolayer cultures for 3 weeks without rAC. At 3 weeks the cells were passaged and re-plated at a density of 1×10^6^/cm^2^, and then grown for an additional 1 week with or without rAC. At the end of this 1-week growth period the cells were analyzed by western blots, revealing that the expression of two chondrogenic markers, aggrecan and Sox9, were highly elevated in the rAC-treated horse cells. Bax expression also was reduced in the rAC cells, suggesting a reduction in apoptosis by rAC treatment. All experiments have been repeated at least 3×. Images are representative from individual experiments.

No effect of rAC treatment on the total number of cells was observed (Fig. 2A), consistent with the fact that the levels of two apoptotic markers, Bax and PARP, also were very similar with and without rAC treatment.

In contrast, there was a marked effect of rAC treatment on the cell quality after 3 weeks of expansion, as determined by the expression of various chondrocyte markers. As shown in Fig. 2B, western blot analysis revealed that the expression of collagen 2A1, collagen IA2, aggrecan, Sox9, TGF-beta1, and FGF2 were enhanced in cells treated with rAC. Similar effects were evident in cells grown using a different culture media, RPMI containing 10% FBS (data not shown). Expression of collagen 10, a marker of chondrocyte hypertrophy and de-differentiation [28], was reduced in these cultures.

To confirm that the effects of rAC were not rat-specific, we studied equine articular chondrocytes provided frozen at P0. The frozen cells were thawed and expanded for 3 weeks without rAC, and then re-plated with or without rAC in the culture media. They were grown for 1 additional week and then analyzed. As shown in Fig. 2C, the expression of two important chondrocyte markers, aggrecan and Sox9, were markedly enhanced in the rAC-treated equine cells, consistent with what was observed in the rat. The lack of other equine-specific antibodies precluded the analysis of additional chondrocyte markers in these cultures. Of interest, we also observed a reduction in the expression of the pro-apoptotic Bax protein in the rAC-treated equine cells, more than that seen in the rat cells. This could be due to the equine cells having been provided to us frozen, eliciting additional stress-related cell death during the culture period that was reduced by rAC.

**Figure 3:**
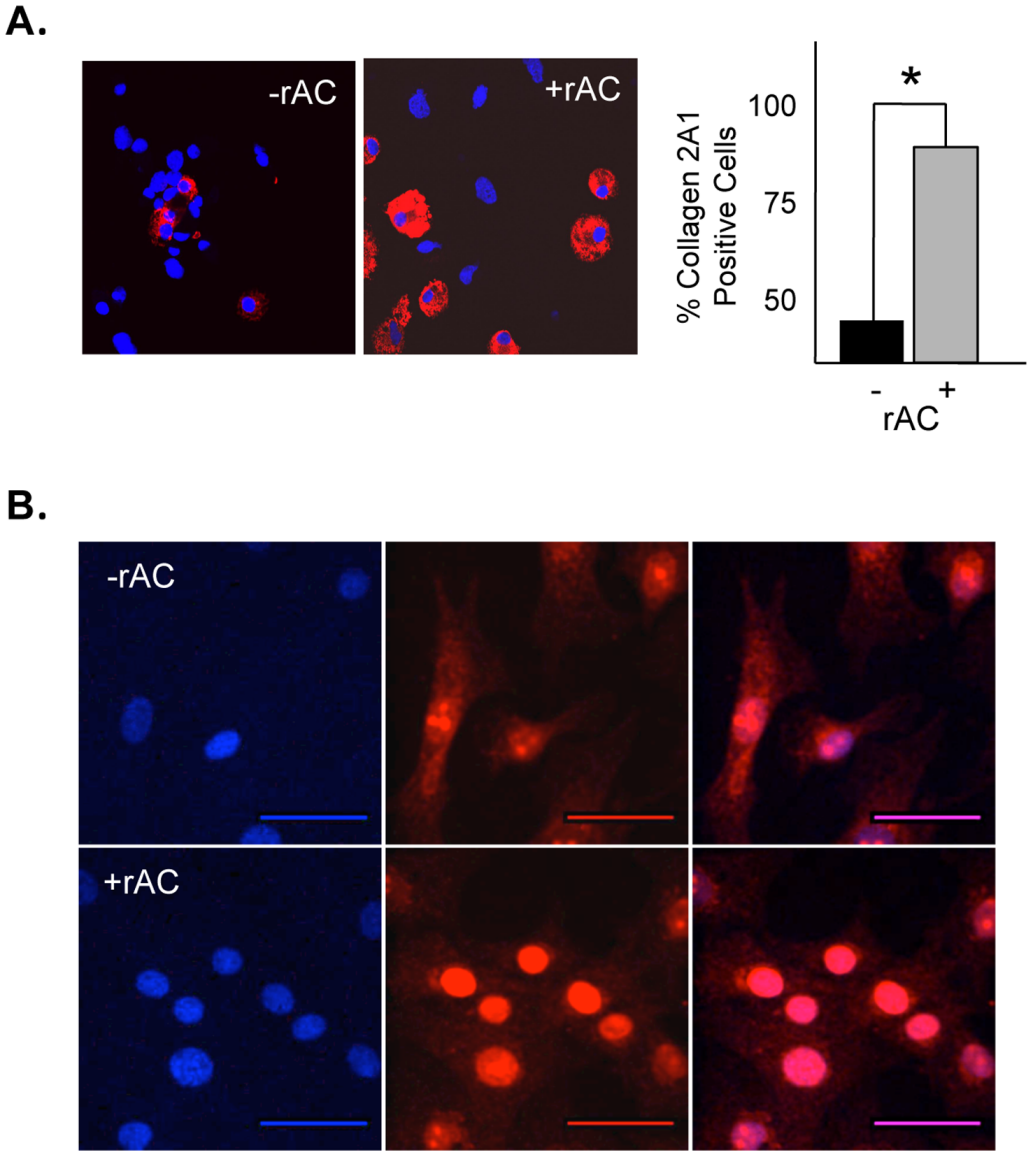
Confocal immunostaining confirming the high level of expression of collagen 2A1 in rat chondrocytes treated with rAC, A) and B) showing different intracellular distribution of Sox9 2 weeks after rAC treatment. Blue (DAPI) indicates nuclei, and red indicates Sox9 expression. Experiments have been repeated at least 3×. Images are representative from individual experiments. Scale bars: 50 µm. * = p<0.05.

We further evaluated rAC-treated and untreated rat chondrocytes by immunohistochemistry and confocal microscopy. These analyses confirmed that after 3 week expansion the number of cells expressing collagen 2A1 was increased ∼40% following rAC treatment (Fig. 3A). In addition, confocal analysis revealed higher levels of Sox9 in the cells after exposure to rAC (Fig. 3B).

**Figure 4:**
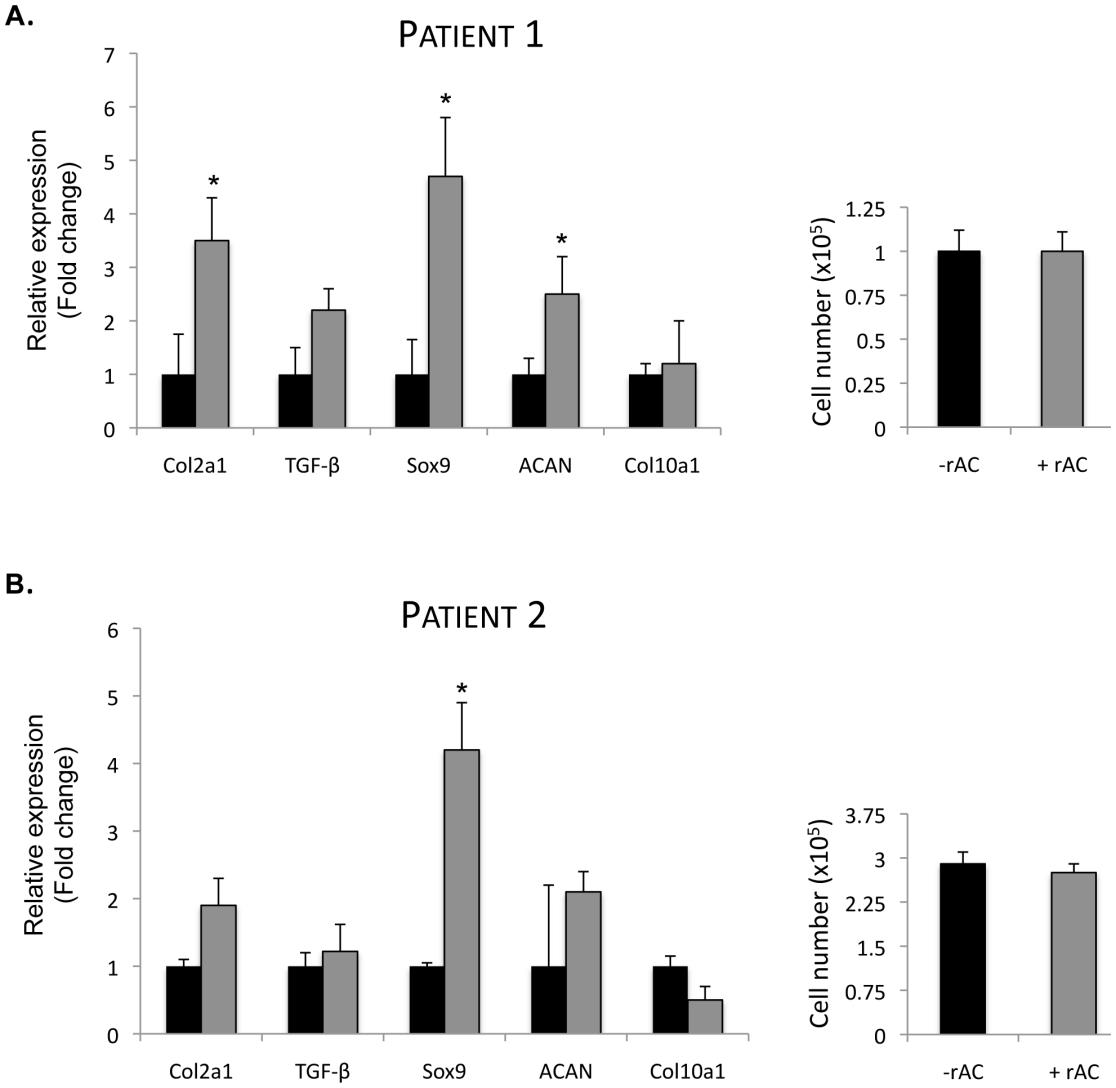
Primary human chondrocytes obtained from the femoral head of a 74-year-old woman with OA were expanded for 3 weeks in monolayer culture with or without rAC added to the culture media (DMEM +10% FBS, rAC added once at the initiation of the culture; 170 U/ml AC). **A)** mRNA expression of several chondrogenic markers (collagen 2A1 [Col2a1], TGF-beta1 [TGF], Sox9 and aggrecan [ACAN]) were analyzed by quantitative RT-PCR. Note the positive influence of rAC on the expression of these markers. The expression of collagen 10 was unchanged in these cultures. **B)** The same experiment was repeated on a second set of cells from a patient with OA. RT-PCR analysis was performed 3×. Consistent with the results obtained with rat and equine chondrocytes (Fig. 2), no significant differences in the number of cells could be found (right panels). * = p<0.05.

Osteoarthritis (OA) is a common, age-related disorder that results in cartilage degradation, and patients with OA frequently undergo surgical procedures to repair their defective joints. Articular chondrocytes from two aged OA patients was obtained and expanded in monolayer culture for 3 weeks with or without rAC in the media (added once at the initial cell plating). As depicted in Fig. 4, qPCR analysis showed that cells from patient 1 exhibited significantly elevated expression of Sox9, collagen 2A1, aggrecan and TGF-beta mRNA in response to rAC treatment. In cells from patient 2, Sox9 expression was significantly elevated, and there was a trend towards elevated collagen 2A1 and aggrecan expression. As was observed with the rat and horse cells, the total number of chondrocytes obtained after the expansion period was similar, despite the differential gene expression pattern. It is again notable that no elevation of collagen 10 expression was observed in the rAC treated cells from either patient.

Overall, these results revealed that supplementing chondrocyte media with rAC once at the time of initial cell plating had a significant, positive effect on the chondrogenic phenotype after expansion.

### Effects of rAC Supplementation on 3-D Culture of Primary Articular Chondrocytes

**Figure 5:**
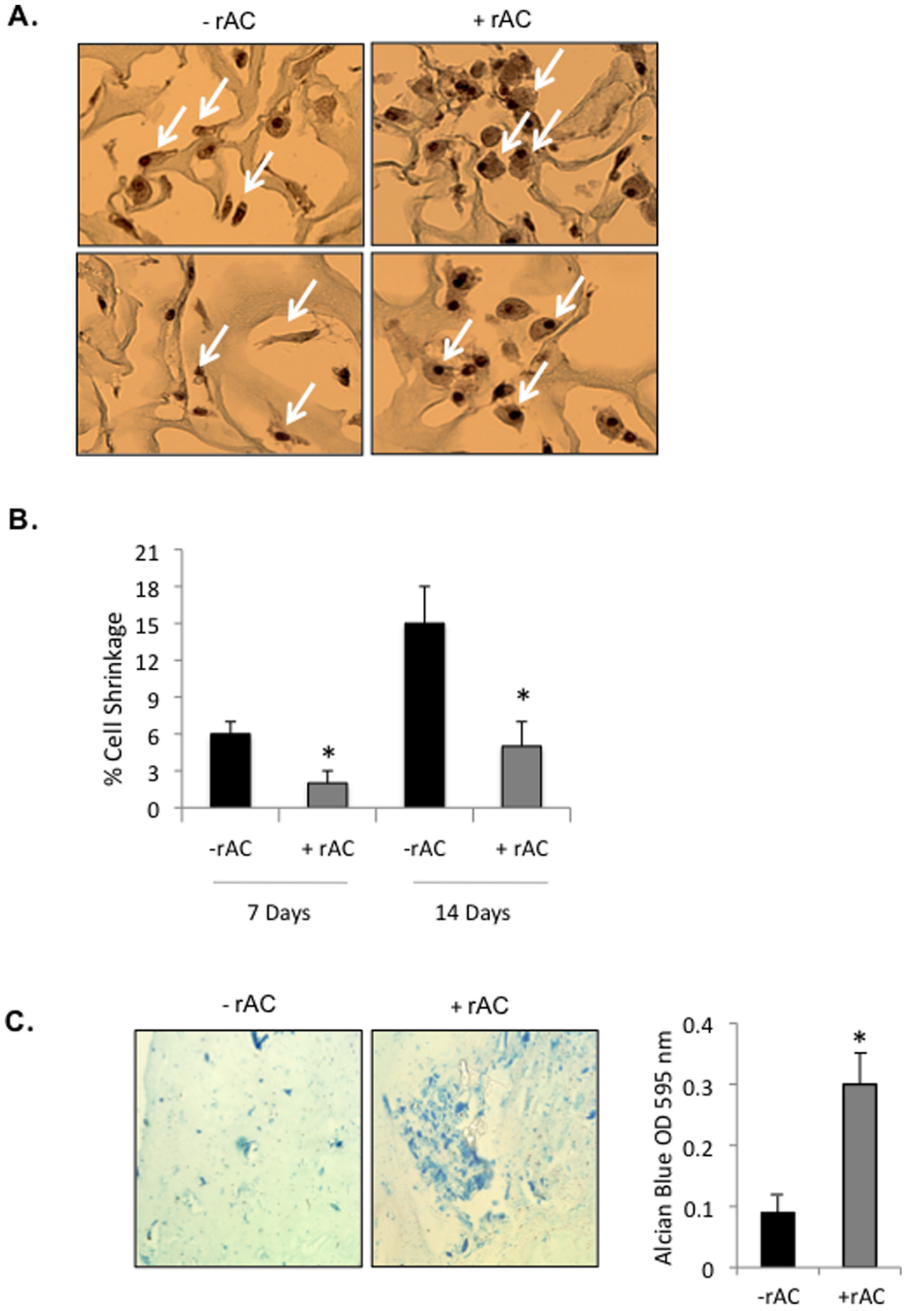
Primary rat chondrocytes were seeded into 3-D collagen scaffolds and grown for 7 or 14 days with or without rAC (DMEM containing 10% serum). **A)** They were then analyzed for morphology and proteoglycan production by Safranin-O staining. Note that the cells grown with rAC were larger and maintained a round phenotype that stained positive with Safranin-O (arrows). Shown are representative images from experiments performed 3×. **B)** Cell shrinkage was evaluated based on Safranin-O and H&E staining using the following equation: DNA unit size = π(fiber segment diameter/2)^2^ × (myofiber segment length)/myofiber nuclei]. * = p<0.05. **C)** Primary rat chondrocytes were grown for 2 weeks in biodegradable fibrin gels with or without rAC in the culture media. Alcian Blue staining, another marker of proteoglycan expression, indicated enhanced chondrogenesis (more intense blue color). Experiment was performed 2×.

To achieve effective implantation of expanded chondrocytes in patients, after the initial monolayer expansion period the cells used for ACI are generally seeded onto 3-D scaffolds for subsequent growth and transplantation. Therefore, the effects of rAC treatment on primary rat chondrocytes grown on collagen-coated scaffolds were evaluated (Fig. 5A). For this experiment, the cells were seeded directly onto the scaffolds bathed in DMEM containing 10% FBS with or without rAC. They were then grown for 7 or 14 days and analyzed. In order to obtain optimal penetration of the 3-D scaffolds with the enzyme, fresh rAC was added to the cells at each media change. As show in the representative images (Fig. 5A), cells grown in the presence of rAC were larger and maintained a rounder phenotype than those without rAC. Importantly, these cells also stained positive with Safranin-O, a commonly used marker of proteoglycan expression. Further quantification of cell shrinkage [29] revealed significantly less shrinkage in the rAC treated cells (Fig. 5B).

In addition to collagen-coated scaffolds, biodegradable fibrin gels also are frequently used for implantation of the cells into the damaged cartilage site. As shown in Fig. 5C, rAC treatment of rat chondrocytes grown in such gels for 2 weeks exhibited enhanced staining for Alcian Blue, an important marker of proteoglycan deposition.

### Effects of rAC Supplementation on the Yield of Bone Marrow-derived Mesenchymal Stem Cells

**Figure 6:**
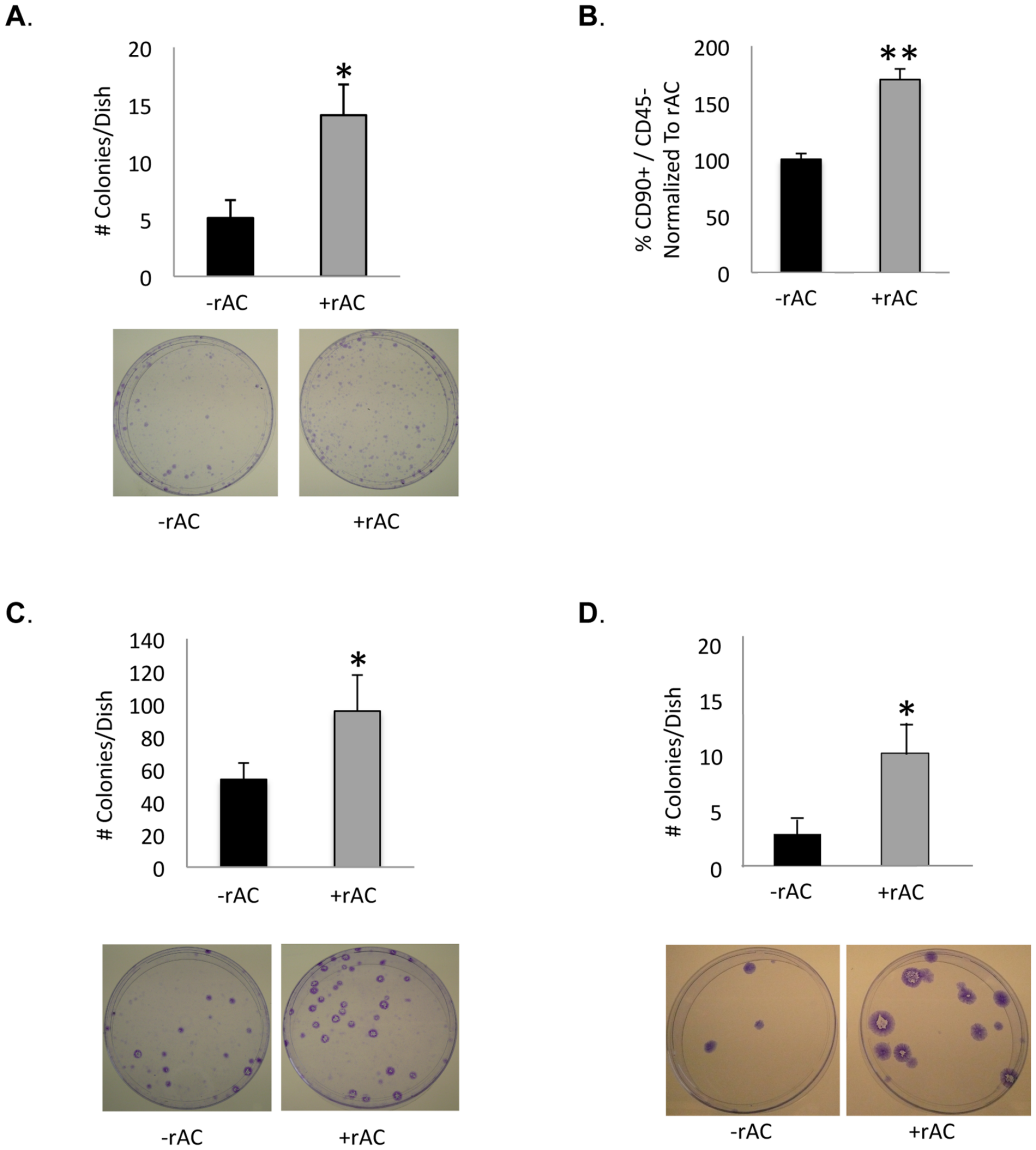
Rat bone marrow cells were isolated and grown for 1 week with or without rAC in standard culture media. rAC was added to the culture media once at the initial plating (5×10E^6^ cells/cm^2^). The number of mesenchymal stem cells (MSCs) in the cultures at 1 week was determined by two assays: **A**) the number of fibroblast-like colony forming units (CFU-F), and **B**) by flow cytometry (CD90+/CD45-). Note that an ∼2–3 fold increase in the number of rat MSCs was observed using rAC. The same result could be reproduced with **C**) feline cells (plated at a density of 1–5×10^6^ cells/cm^2^) and **D**) equine cells (plated at 3×10^8^ cells/cm^2^). * = p<0.05, ** = p<0.005. Representative culture plates showing the stained colonies are provided below the quantitative data.

As noted above, recent interest in cell-based cartilage repair has focused on the use of MSCs, which can be readily obtained from adult bone marrow, adipose tissue or other autologous sources, and may be induced *in vitro* or *in vivo* to form chondrocytes and other mesenchymal cell lineages. The effects of rAC on adult rat bone marrow-derived MSCs before and after their differentiation into chondrocytes were therefore evaluated. Addition of rAC to the culture media at the time of initial plating of the rat bone marrow cells led to a ∼2–3 fold increase in the number of MSCs obtained at day 5, as judged by the number of colony forming fibroblast units (CFU-F) or by flow cytometry (Fig. 6A,B). To confirm that these findings were not rat-specific, the effect of rAC supplementation on bone marrow MSCs obtained from cats and horses also was evaluated (Fig. 6C,D). Due to the lack of suitable antibody reagents for flow cytometry, the number of MSCs in these species were quantified by CFU-F only, revealing increases similar to those observed with the rat cells.

### Effects of rAC Supplementation on the Chondrogenic Differentiation of Bone Marrow Mesenchymal Stem Cells

**Figure 7:**
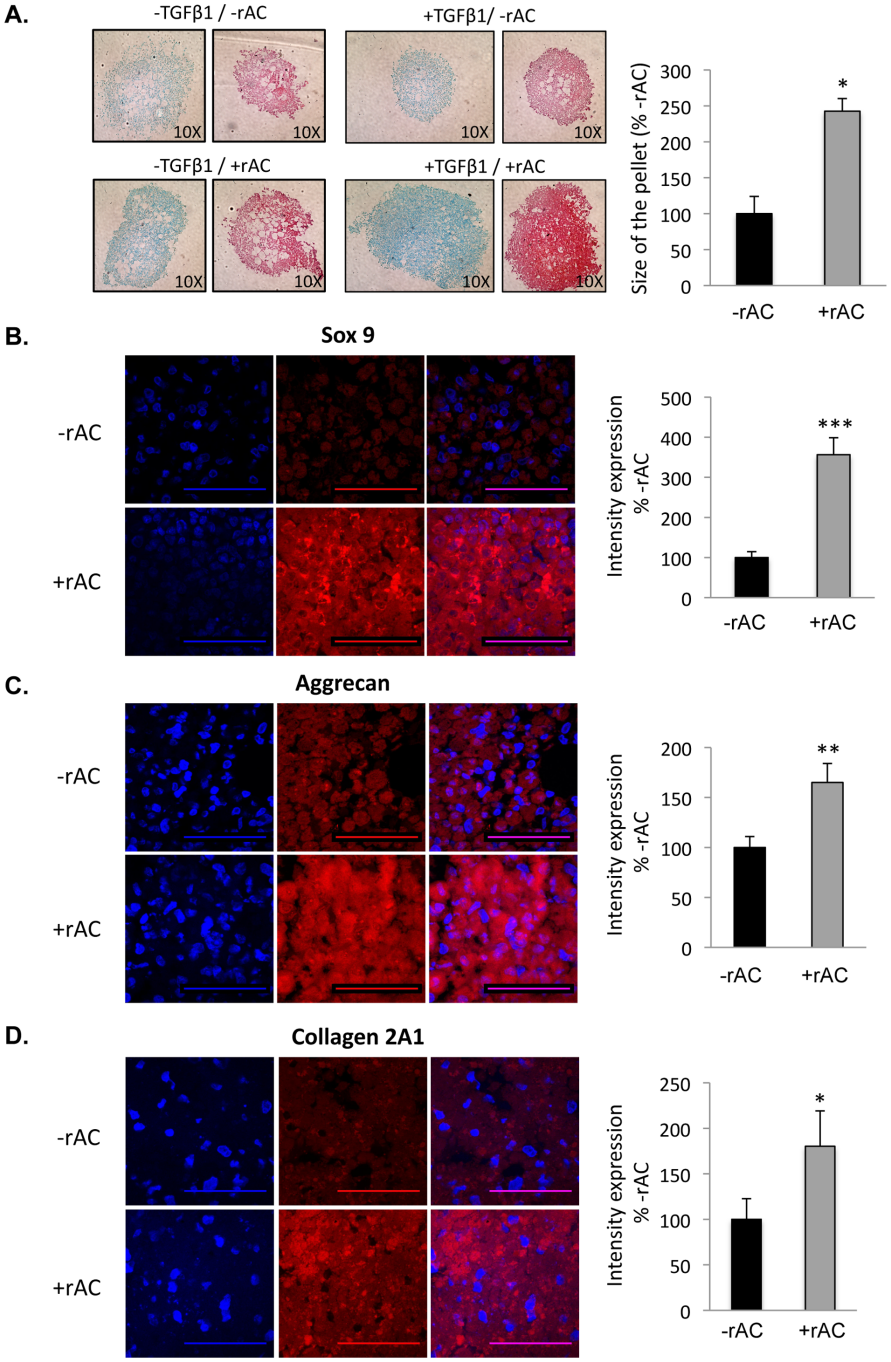
Rat bone marrow cells were isolated and expanded for 3 weeks in standard culture media (without rAC) to prepare homogenous populations of MSCs. They were then placed into chondrocyte differentiation media (Stem Cell Technology) with or without TGF-beta1 and/or rAC. Pellet cultures were grown for 3 weeks to prepare chondrocytes, and then fixed and analyzed by Alcian Blue and Safranin-O staining, markers of chondrogenesis. **A)** Note the small and poorly formed pellets in the absence of TGF-beta1 and rAC (upper left). TGF-beta1 is a standard supplement used to induce the differentiation of bone marrow MSCs to chondrocytes. Addition of TGF-beta1 or rAC to the cultures independently had only a modest effect on the pellet size and staining (upper right and lower left). However, inclusion of both proteins in the culture media had a much more significant effect (lower right), both on the size of the pellets and staining intensity. Pellet size is an indicator of the number and/or size of chondrocytes, and staining intensity is a measure of proteoglycan deposition. Beside Alcian Blue and Safranin-O staining, pellets were subjected to immunostaining against **B)** Sox9, **C)** Aggrecan, and **D)** Collagen 2A1. Experiments have been performed with 3 independent rats. Representative images are shown from one experiment. Blue (DAPI) indicates nuclei, and red indicates Sox9, Aggrecan or Collagen 2A1, respectively. Merged images are to the right. Scale bars: 50 µm. * = p<0.05, ** = p<0.005, *** = p<0.001.

**Figure 8:**
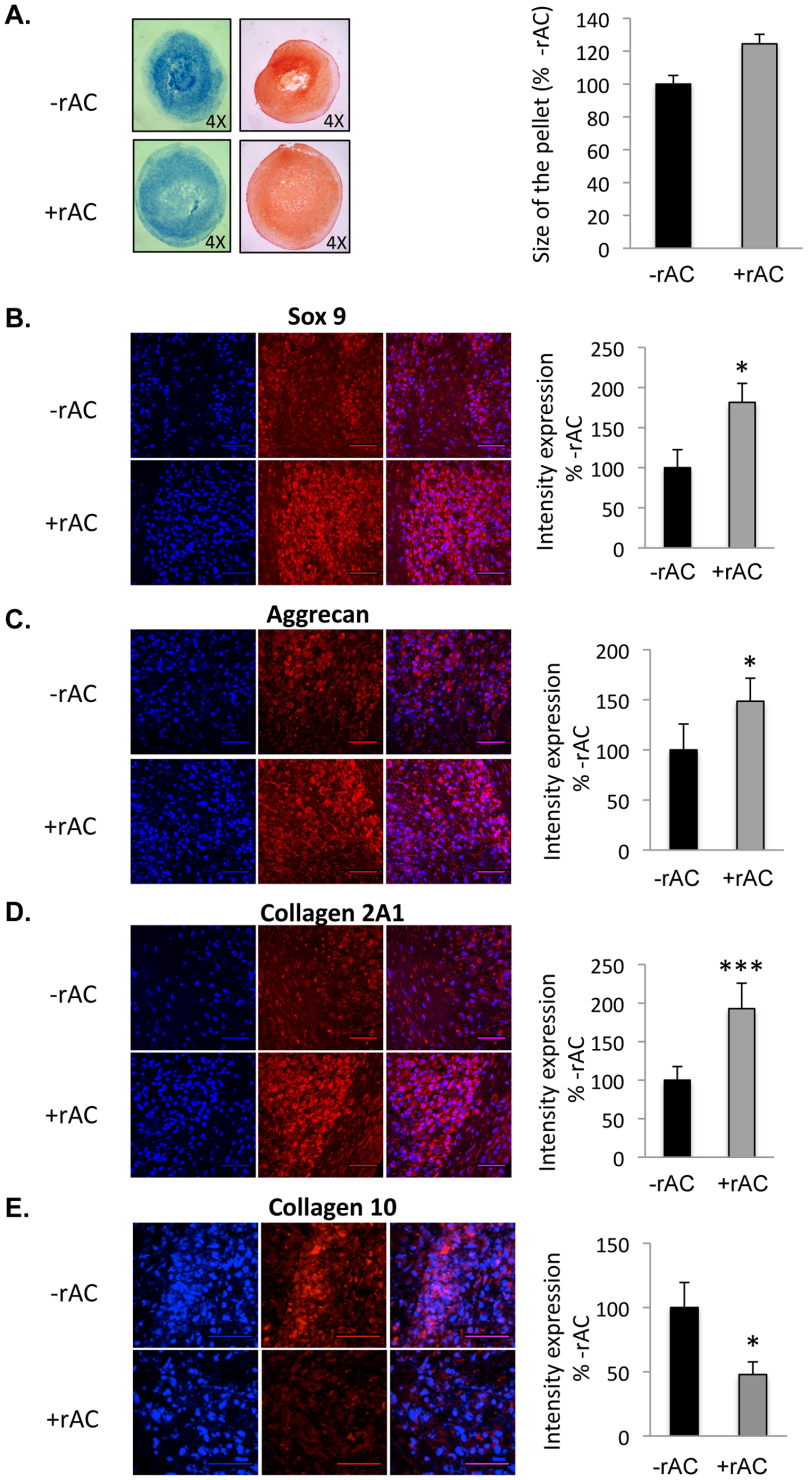
Horse bone marrow cells were isolated and expanded for 3 weeks in standard culture media (without rAC) to prepare homogenous populations of MSCs. They were then placed into chondrocyte differentiation media containing TGF-beta1, but with or without rAC. **A)** Pellet cultures were grown for 3 weeks to prepare chondrocytes, and then fixed and analyzed by Alcian Blue (left panel) and Safranin-O (right panel) staining. Note the smaller, more diffuse pellets in the absence of rAC. Pellets were also submitted to immunostaining against **B)** Sox9, **C)** Aggrecan, or **D)** Collagen 2A1. Note the higher expression intensity of Sox9, Aggrecan and Collagen 2A1 in the pellets. **E)** Pellets were also subjected to an immunostaining against Collagen 10. Note the diminished expression of Collagen 10 in pellets exposed to rAC. DAPI (blue left panel) indicates nuclei, and the right panel (red) indicates Sox9, Aggrecan, Collagen 2A1, or Collagen 10, respectively. Merged images are to the right. Scale bars: 50 µm. * = p<0.05, ** = p<0.005, *** = p<0.001.

The effects of adding rAC during the chondrogenic differentiation of MSCs also were evaluated. For these studies differentiation was carried out in high-density pellet cultures using standard media with or without TGF-beta1 (see Materials and Methods). Rat bone marrow cells were first grown for 3 weeks in the absence of rAC to obtain a population of adherent MSCs that was ∼90% pure (CD90+/CD40-, not shown). They were then grown in high-density pellet cultures for an additional 3 weeks in chondrocyte differentiation media. As shown in Fig. 7A, rat pellets grown in the presence of rAC were larger (>2-fold) and stained more intensely for proteoglycans using Alcian Blue and Safranin-O. The effects of rAC were independent of TGF-beta1, and the two factors worked synergistically to yield optimal results. The rat pellets also were analyzed by immunohistochemistry and confocal microscopy for Sox9, aggrecan and collagen 2 expression. As illustrated by the images in Figs. 7B-D, the expression of these three chondrogenic markers were significantly elevated in cells supplemented with rAC. To confirm these findings, the same studies were performed using equine cells, and essentially the same results were obtained (Fig. 8A–D).

As described earlier for the chondrocyte expansion studies, rAC treatment led to either no change or a reduction in the levels of collagen 10, a marker of chondrocyte hypertrophy and de-differentiation, in contrast to collagen 2, which was elevated. To examine this further the differentiated equine MSC-derived chondrocytes were stained for collagen 10, and the levels were found to be decreased in cells treated with rAC (Fig. 8E). Taken together, the elevated expression of collagen 2, aggrecan and Sox9 along with low collagen 10 expression suggests that the treated cells had a more chondrogenic phenotype conducive to cartilage repair.

## Discussion

The dedifferentiation of chondrocytes during cell expansion remains one of the main barriers in ACI [30]. During the amplification process it takes only a few days for the cells to change their shape from rounded to fibroblastic and to start displaying abnormal features. For example, the cytoskeleton begins to express F-actin stress-fibers, important chondrocyte-specific extracellular matrix proteins and/or transcription factors are reduced (e.g., Col 2, proteoglycans, Sox9), hypertrophic makers (e.g., Col 10) may become elevated, and degradative proteases (e.g., MMPs, ADAM-TS) are activated. Moreover, although dedifferentiated cells can be re-differentiated when implanted in 3-D culture systems such as alginate, agarose or fibrin hydrogels, irreversible de-differentiation is usually observed in preparations of primary chondrocytes. Hence, during the expansion phase it is of primary interest to maintain primary chondrocytes as close as possible to their initial chondrogenic profile, and/or to improve their capacity to re-differentiate prior to re-implantation.

The current study shows that a single treatment of primary chondrocytes with rAC, although not improving the yield of cells after expansion, greatly improved their chondrogenic phenotype. In particular, the expression of collagen 2A1, aggrecan, FGF2 and Sox9 were markedly increased in rat cells at the end of the 3-week expansion period (Figs. 2 and 3). Similar findings were observed in equine and human cells (Figs. 2 and 4). When grown in 3-D culture systems, i.e. collagen sponges or fibrin gels, addition of rAC also improved the cell phenotype, as shown by H&E and Safranin-O staining (data not shown and Fig. 5). Cells were bigger and rounder, and the expression of proteoglycans was improved with rAC treatment (Fig. 5C).

It may be speculated that these changes were due to the short-term effects of rAC treatment on sphingolipid metabolism (Fig. 1), presumably leading to transcriptional and post-translational modifications that resulted in these downstream effects. rAC activity was rapidly elevated in primary chondrocytes after addition of the enzyme to the culture media, lowering the levels of ceramide compared to controls, and resulting in the elevation of sphingosine by 48 h. Ceramide is a well characterized, stress-response lipid that has many effects on cell survival and differentiation. Sphingosine, the product of AC activity, has similar biological effects, and both of these lipids have been shown to influence chondrogenesis. For example, ceramide may stimulate aggrecanse activity, leading to proteoglycan depletion in cells [13]. On the other hand, ABC29460, a selective inhibitor of sphingosine kinase 2, an enzyme that converts sphingosine to S1P, attenuated cartilage damage and reduced chondrocyte apoptosis in a rat model of osteoarthritis [16]. This suggests that sphingosine may have a protective effect on chondrocytes. The role of S1P itself also has been studied extensively in cultured chondrocytes and in animal models of OA with somewhat conflicting results; in some cases S1P has been shown to reduce aggrecan and proteoglycan expression [31], while in others it has a protective effect [32]. Clearly, sphingolipids have important effects on chondrocyte growth and differentiation, and manipulation of this pathway with rAC appears to be maintaining the cells in a more chondrogenic phenotype after expansion. Future research should focus on the early transcriptional and other changes occurring in chondrocytes treated with rAC to better understand the mechanism of these positive, downstream effects on the chondrocyte phenotype.

In addition to the use of primary chondrocytes for ACI, many investigators have turned to MSCs for cartilage repair. The main limitations in the use of MSCs for cell-based cartilage repair procedures are that they lose their ability to differentiate into chondrocytes very early (3 or 4 passages), shortening the time for amplification prior to differentiation, and proper *ex vivo* and *in vivo* protocols to differentiate MSCs into fully functional chondrocytes that do not undergo hypertrophy are still lacking. Numerous papers have been published describing the use of new agents for the differentiation of MSCs towards chondrocytes. Among the various molecules evaluated, proteins of the TGF-beta1 and bone morphogenic families (BMPs) are the most described. Another strategy relies on the engineering of 3-D scaffolds that release growth factors, anti-apoptotic or differentiation factors in which the MSCs can be seeded at high density to mimic the natural conditions of differentiation.

In this study a single addition of rAC into the culture media of rat bone marrow was shown to increase the yield of MSCs at 1 week (Fig. 6), as seen by CFU-F assay and flow cytometry analyses. Repeated treatment with rAC during the chondrogenic differentiation process also improved the quality of resulting MSC-derived chondrocytes, as seen by Alcian blue staining, Safranin-O staining, and immunostaining (Figs. 7 and 8). Notably, the levels of Sox9, collagen 2A1 and aggrecan were more elevated in cells differentiated in the presence of rAC, but the level of collagen 10 was diminished, revealing a partial blockage of hypertrophy in cells differentiated in presence of rAC. Reduced collagen 10 also was observed in primary chondrocytes treated with rAC.

Thus, two positive influences of rAC treatment were observed with MSCs, although the mechanisms behind these observations might be different. For example, while the treatment of bone marrow cells with rAC at P0 may have been beneficial because the enzyme reduced the ceramide-related stress response induced by the extraction of the cells from their natural environment and plating in an artificial medium (thereby yielding more viable MSCs), the benefit of the treatment during the differentiation process may be due to other mechanisms such as better signaling through the TGF-beta pathway and/or effects of sphingolipid changes on chondrogenesis and cartilage homeostasis. In addition, reduction of the stress response during MSC chondrodifferentiation may be a factor, in particular since the differentiation medium is devoid of serum and hence a very potent stress inducer.

Overall, we show for the first time that the addition of rAC to culture media has a positive influence on the chondrogenic phenotype of expanded primary and MSC-derived chondrocytes, likely through alterations of the sphingolipid signaling pathway. These findings could have an important impact on cell-based cartilage repair by providing higher quality cells for transplantation, and/or by including the enzyme directly in 3-D scaffolds to improve chondrogensis *in vivo*. Future studies will focus on evaluating the effects of rAC treated cells *in vivo*, as well as exploring the mechanisms underlying these AC-induced changes more fully.
